# TLR ligand sensing by lymph node FRCs directs intranodal lymphocyte accumulation to promote immune responses

**DOI:** 10.1016/j.isci.2025.113734

**Published:** 2025-10-08

**Authors:** Antonio P. Baptista, Eelco Keuning, Reina E. Mebius

**Affiliations:** 1 Department of Molecular Cell Biology and Immunology, Amsterdam University Medical Center, Location VUmc, Amsterdam, the Netherlands; 2 GABBA PhD Program, University of Porto, Porto, Portugal; 3 Laboratory of Immunoregulation and Mucosal Immunology, VIB Center for Inflammation Research, Ghent, Belgium; 4 Department of Internal Medicine and Pediatrics, Ghent University, Ghent, Belgium

**Keywords:** biological sciences, immunology

## Abstract

Immune protection depends on antigen-responsive lymphocytes finding their cognate antigen on competent antigen-presenting cells. To increase the likelihood of such an event happening, lymphocytes transiently accumulate in secondary lymphoid organs soon after infection. Here, we show that this phenomenon requires expression of Toll-like receptors (TLRs) on lymph node stromal cells. Direct sensing of pathogen-associated molecular patterns by TLR-expressing fibroblastic reticular cells (FRCs) rapidly induced homeostatic chemokine expression, mediating immediate lymphocyte accumulation into reactive lymph nodes. Ablation of this response, by means of *Tlr4*^−/−^ lymph node transplantation or conditional *Tlr4* gene deletion on PDGFRb^+^ cells, reduced the number of lymphocytes recruited into the immune response limiting vaccine efficacy against tumors. Taken together, these observations provide further evidence for a critical role of early FRC activation in driving effective immunity, placing these non-hematopoietic cells at the center of adaptive host protection.

## Highlights

• LPS exposure triggers rapid, CCR7-dependent intranodal lymphocyte accumulation
• Lymph node FRCs respond to LPS exposure rapidly with increased CCL19/CCL21 production
• FRC responses to LPS mediate intranodal lymphocyte accumulation
• FRC responses to LPS power T cell responses quantitatively

## Graphical abstract

**Figure:**
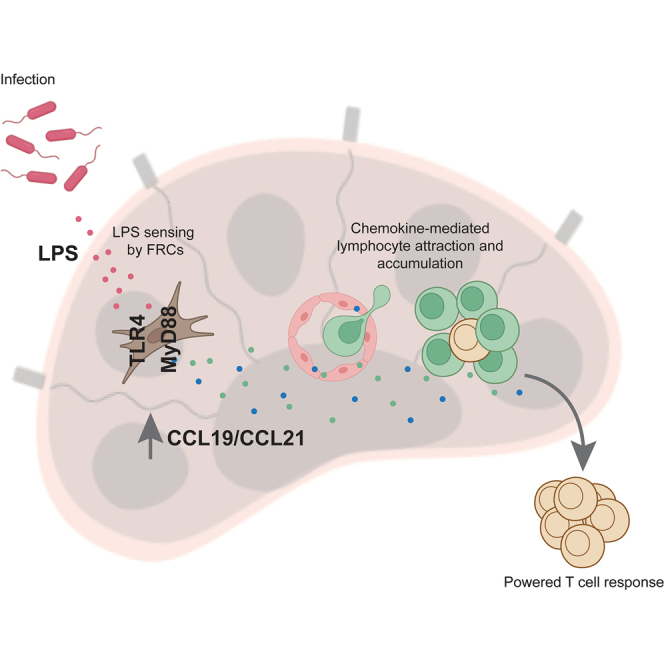

## Introduction

Effective protection against disease relies on the timely and robust activation of antigen-specific lymphocytes by competent cognate antigen-presenting cells (APCs). Both of these cells are very rare early after infection, with estimates of naive precursor frequency suggesting the number of T cells specific for any given foreign antigen to be in the range of fewer than 10 to a few hundred cells per mouse,^1^^,^^2^^,^^3^ with comparable scaling for humans.^1^^,^^4^ We and others have previously shown that such quantitative challenges are partially overcome by concentrating both cell types as well as antigens in secondary lymphoid organs,^5^^,^^6^^,^^7^ and by the presence of intricate spatial arrangements within these organs that preemptively favor pathogen/antigen recognition.^8^^,^^9^^,^^10^^,^^11^^,^^12^^,^^13^^,^^14^^,^^15^ Despite these strategies, the sparseness of antigen-specific T cells and antigen-carrying APCs in secondary lymphoid organs is likely to remain a significant hurdle to the development of anti-pathogenic responses unless additional mechanisms are in place to facilitate antigen detection.

These quantitative challenges manifest themselves in, at least, two critical ways. First, the likelihood of timely antigen detection. Second, the probability of the induced immune response to be properly tailored, as T cell effector differentiation at the single cell level seems to be a random process.^16^^,^^17^^,^^18^ Both issues can only be solved by recruiting sufficiently large numbers of naive T cell precursors into the immune response through which the breadth and thus the probability of developing a well fitted effector response will increase.

In the steady-state, lymphocyte migration to, within and from lymph nodes (LNs) is regulated by lymphoid stromal cells (SCs) through the production of multiple adhesion and chemoattractant molecules.^5^ Relevant to APC-T cell communication, lymphoid SC networks work both as anchorage sites for conventional dendritic cells (cDCs)^19^ as well as local roads defining the paths of lymphocyte intranodal migration.^20^ Considering both these data and the requirement for antigen recognition around specialized LN environments defined by SCs,^8^^,^^21^^,^^22^^,^^23^ it is likely that lymphoid SCs may be directly involved in the sudden, non-antigen-driven, increase in LN cellularity observed early after infection/immunization^24^^,^^25^^,^^26^^,^^27^^,^^28^ and hence may actively contribute to the developing immune response. Notwithstanding, the available evidence suggest that infection limits the SC ability to sustain immune cell recruitment.^29^^,^^30^^,^^31^ Such phenomenon is not only incompatible with LN expansion seen in most infection settings, but more importantly it directly contradicts data suggesting that LN SC activation is critical to intranodal B and T cell responses.^32^^,^^33^^,^^34^^,^^35^^,^^36^^,^^37^ Here, we show that LN fibroblastic reticular cells (FRCs) directly and acutely responded to stimulation with pathogen components with increased chemokine expression. Among the increased chemokines, CCL19 and CCL21 enhanced lymphocyte accumulation within reactive LNs, augmenting the number of T cells enlisted into the immune response, and thus effectively contributing to the efficacy of an anti-tumor vaccine. Taken together, our findings suggest that homeostatic lymphocyte recirculation patterns can be rapidly adjusted to concentrate lymphocytes in the LNs draining sites of infection, placing FRCs at the center of adaptive T cell responses.

## Results

### LPS directs lymphocyte accumulation in lymph nodes via increased homeostatic chemokine expression

**Figure 1:**
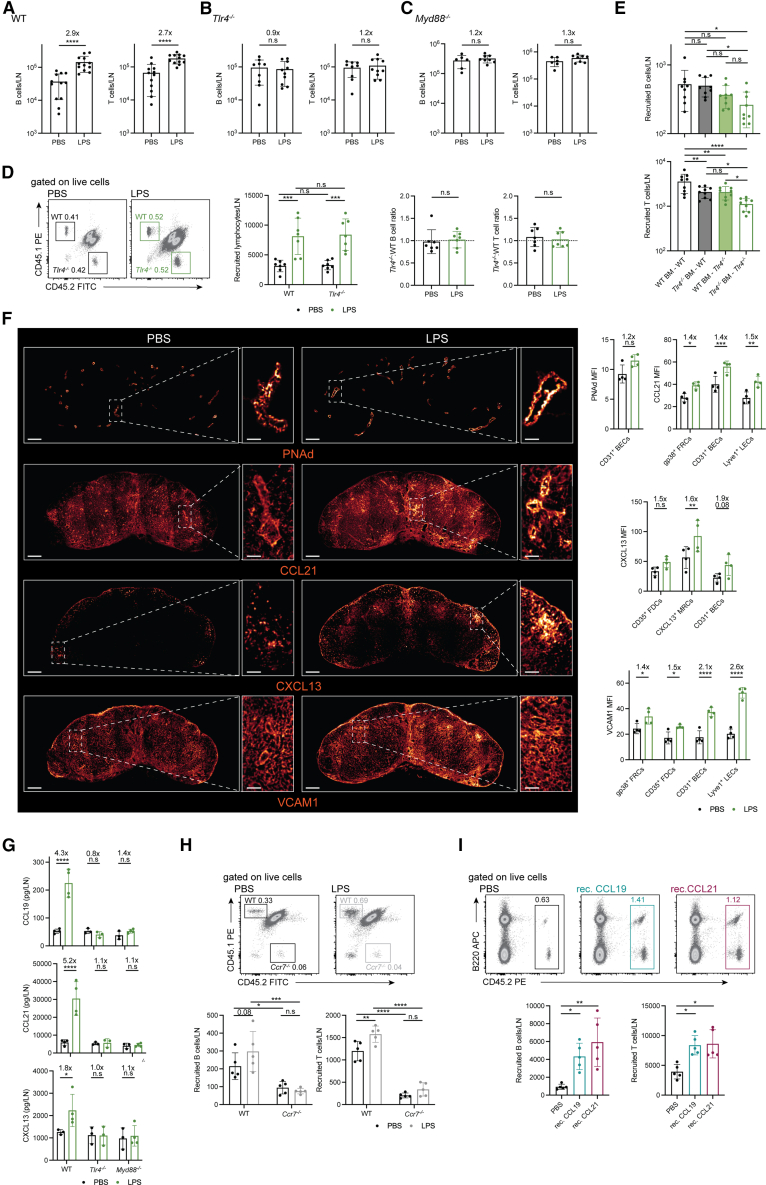
LPS directs intranodal lymphocyte accumulation via increased CCL19/CCL21 expression (A–C) Lymphocyte numbers in the popliteal LNs of WT (A), *Tlr4*^−/−^ (B) or *Myd88*^−/−^ (C) mice 6 h after s.c. injection of PBS or LPS. *n* = 12 WT mice/group, 9–10 *Tlr4*^−/−^ mice/group, 6–8 *Myd88*^−/−^ mice/group. Unpaired *t* test. Numbers at the top of the graphs indicate fold changes in cellularity as compared to PBS injections. (D) Representative FACS plots, number of *de novo* accumulating lymphocytes, and competitive B and T cell ratios between CD45.1^+^ WT and CD45.2^+^*Tlr4*^−/−^ lymphocytes recovered from the popliteal lymph nodes of CD45.1^+^CD45.2^+^ WT recipients 6 h after *i.v* cell injection, and PBS or LPS *s.c* administration. *n* = 7 mice/group. two-way ANOVA with Sidak’s multiple comparisons post-test (left) and unpaired *t* test (right). (E) Number of *de novo* accumulating CD45.1^+^ WT B cells and T cells recovered from the popliteal lymph nodes of WT:*Tlr4*^−/−^ chimeric mice 6 h after *i.v* cell injection and LPS *s.c* administration. *n* = 9 mice/group. one-way ANOVA with Tukey’s multiple comparisons post-test. (F) Representative immunofluorescence images and quantification of intranodal PNAd, CCL21, CXCL13 and VCAM1 expression on lymph node stromal cells, 6 h after PBS or LPS administration to WT mice. Signals are depicted in a divergent scale of red to yellow. Scale bar, 200 μm; insets = 50 μm. *n* = 4 mice/group. Two-way ANOVA with Sidak’s multiple comparisons post-test. Numbers at the top of the graphs indicate fold changes in MFI expression as compared to PBS injections. (G) Intranodal CCL19, CCL21, and CXCL13 expression as quantified by ELISA, 6 h after PBS or LPS administration to WT, *Tlr4*^−/−^ or *Myd88*^−/−^ mice. n = 3–4 mice/group. Two-way ANOVA with Sidak’s multiple comparisons post-test. (H) Representative FACS plots and number of *de novo* accumulating CD45.1^+^ WT and CD45.2^+^*Ccr7*^−/−^ lymphocytes recovered from the popliteal lymph nodes of CD45.1^+^CD45.2^+^ WT recipients 6 h after *i.v* cell injection, and PBS or LPS *s.c* administration. *n* = 5 mice/group. Two-way ANOVA with Sidak’s multiple comparisons post-test. (I) Representative FACS plots and number of *de novo* accumulating CD45.1^+^ WT lymphocytes recovered from the popliteal lymph nodes of CD45.2^+^ WT recipients 6 h after *i.v* donor cell injection, and PBS, recombinant CCL19 or recombinant CCL21 *s.c* administration. *n* = 5 mice/group. one-way ANOVA with Dunn’s multiple comparisons post-test. Data represent the mean ± SD with superimposed individual data points. n.s, not significant; ∗, *p* < 0.05; ∗∗, *p* < 0.01; ∗∗∗, *p* < 0.001; ∗∗∗∗, *p* < 0.0001.

Lymphocytes rapidly accumulate in secondary lymphoid organs following infection. To mimic this event in an infection-free environment, we administered lipopolysaccharide (LPS), a component of the cell wall of Gram-negative bacteria, to mice, which caused rapid B and T cell accumulation in lymph nodes (LNs) within 6 h after *s.c* injection, mediated by signaling through Toll-like receptor 4 (TLR4) and MyD88 (Figures 1A–1C). Notably, while previous reports indicate a role for type I interferons (IFNs) in trapping lymphocytes in LNs upon TLR triggering,^38^^,^^39^^,^^40^^,^^41^^,^^42^ analysis of *Ifnar1*^−/−^ mice revealed only a partial inability of lymphocytes to accumulate into reactive LNs within this time frame (Figure S1A), suggesting that in our experimental conditions acute intranodal lymphocyte accumulation is regulated by additional mechanisms. To better explore the basis of LPS-driven lymphocyte accumulation into reactive LNs, we devised a strategy based on the injection of congenically marked cells to quantify *de novo* accumulating cells. First, we verified that the transferred cells accumulated more readily in LPS-exposed as compared to PBS-exposed LNs (Figure 1D), validating our approach. LPS could still induce intranodal lymphocyte accumulation when the entry of lymphocytes into lymph nodes was blocked with anti-CD62L antibodies (Figure S1B); and LPS could still promote lymphocyte accumulation when FTY720 prevented lymphocyte egress (Figure S1C). Taken together, these results suggested that LPS could independently modulate lymphocyte entry into and egress from reactive lymph nodes. Additionally, when WT and *Tlr4*^−/−^ lymphocytes were simultaneously transferred, our strategy showed that the intrinsic ability of lymphocytes to recognize and respond to LPS was dispensable for their intranodal retention (Figure 1D); and when applied to bone marrow chimeric mice, it indicated a role for both hematopoietic and non-hematopoietic TLR4 expression in directing intranodal lymphocyte accumulation, particularly evident for T cells (Figure 1E). Hence, LPS seemed to interfere with lymphocyte recirculation dynamics upon being sensed by both non-lymphoid hematopoietic cells and radioresistant SCs.

Whereas acute inhibition of lymphocyte egress from lymph nodes in response to TLR ligands is relatively well understood,^42^ enhanced lymphocyte recruitment at the early time points we report on has received little attention. Lymphocyte recruitment/accumulation in tissues is determined by engagement of multiple adhesion and signaling receptors.^5^ To gain insights into the entry mechanisms modulated by LPS, we analyze the intranodal abundance of transcripts for molecules involved in leukocyte tissue migration. Whereas no consistent changes in the expression of transcripts involved in early tethering and rolling (scaffold molecules and enzymes—*Podxl*, *Glycam1* and *Chst4*—regulating peripheral-node addressing (PNAd) expression) were noted, mRNA transcripts for several chemokines and adhesion molecules involved in lymphocytes’ firm adhesion to the endothelium, transmigration, and tissue accumulation increased upon LPS exposure in a TLR4-dependent manner (Figures S1D–S1F). Consistent with these transcriptional changes, protein quantification via immunofluorescence imaging coupled to histocytometry^12^ (Figure S1G) revealed no significant differences in PNAd staining intensity, but a rapid upregulation of CCL21, CXCL13, and VCAM1 in multiple SC (SC) subsets including on high endothelial venules (Figure 1F), the spatial point for lymphocyte transmigration into the LN parenchyma.^5^ CCL21 and CXCL13 upregulation in SCs was TLR4- and MyD88-dependent (Figures S1H and S1I). Taken together, these RNA and protein analyses suggested a direct role for chemokines and adhesion molecules in inducing stronger lymphocyte arrest on and transmigration across blood vessels into the LN parenchyma upon LPS exposure.

Among the changes observed, only modulation of homeostatic chemokine expression paralleled a requirement for the adaptor molecule MyD88 in driving intranodal lymphocyte accumulation in response to LPS (Figures S1F and S1I). Therefore, we posited that homeostatic chemokines may be responsible for the immediate attraction to and/or retention of lymphocytes within reactive LNs. Given the discrepancies in *Ccl21* mRNA and CCL21 protein measurements observed, however, we decided to further validate our data with ELISA. Analysis of lymph node homogenates by ELISA indicated that LPS readily induced CCL19, CCL21, and CXCL13 expression in a TLR4- and MyD88-dependent manner at 6 h after LPS administration (Figure 1G). Lymphocyte migration into LNs in the steady-state is regulated by a CCL19/21-CCR7 axis. Hence, to directly probe an additional role for CCL19 and CCL21 in redirecting lymphocytes toward LPS-exposed LNs, we employed co-adoptive transfers of WT and *Ccr7*^−/−^ splenocytes into WT recipients. Whereas WT lymphocytes showed enhanced accumulation in LPS-exposed LNs as compared to steady-state nodes, in the same recipients, *Ccr7*^−/−^ lymphocytes failed to show increased trafficking into reactive nodes, remaining meagerly represented (Figure 1H). These results showed that CCR7, and by inference CCL19 and/or CCL21, were necessary players in rapidly redirecting lymphocytes toward reactive LNs upon LPS administration. To determine if these factors were also sufficient, we quantified lymphocyte accumulation into LNs exposed to recombinant CCL19 or CCL21 by *s.c* injection. Both recombinant chemokines readily increased the recovery of transferred lymphocytes (Figure 1I). Collectively, our data reveal an essential, non-redundant role for rapid intranodal upregulation of homeostatic chemokine expression in driving immediate lymphocyte accumulation into reactive LNs.

### Direct sensing of LPS by lymph node stromal cells drives lymphocyte accumulation

**Figure 2:**
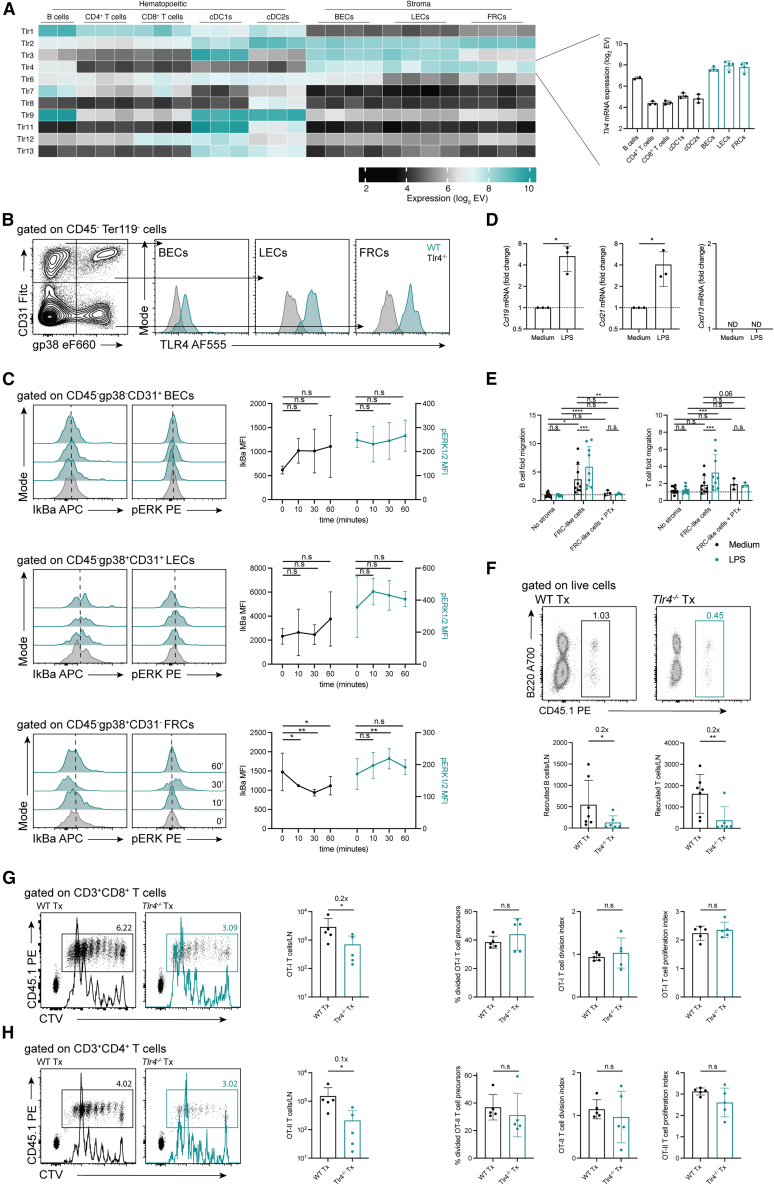
TLR4 expression in lymph node stromal cells directs intranodal lymphocyte accumulation (A) Heatmap analysis of *Tlr* mRNA transcript expression by cells sorted from skin-draining lymph nodes of WT mice as determined by microarray analysis (log2-transformed data). *Tlr4* mRNA expression highlighted in the bar graph on the right. (B) Representative FACS plots of TLR4 expression on gp38^−^CD31^+^ blood endothelial cells (BECs), gp38^+^CD31^+^ lymphatic endothelial cells (LECs), and gp38^+^CD31^−^ fibroblastic reticular cells (FRCs) of WT and *Tlr4*^−/−^ origin. *n* = 3 independent experiments. (C) Representative FACS plots and quantification of IkBa and pERK expression on WT BECs, LECs, and FRCs stimulated *ex vivo* with LPS for the indicated amounts of time. *n* = 4 mice. Two-way ANOVA with Dunnet’s multiple comparisons post-test. (D) Homeostatic chemokine mRNA transcript levels in FRC-like stromal cell lines of WT origin cultured in the presence of LPS for 6 h. Fold changes are calculated over untreated conditions (dotted line). *n* = 3 independent repeats. Unpaired *t* test. N.D, not detected. (E) Quantification of lymphocyte *in vitro* migration across Transwell membranes toward WT FRC-like stromal cell lines cultured in the presence of LPS. Fold change is calculated over lymphocyte automigration in conditions without stroma and without LPS. *n* = 3 independent experiments with triplicate conditions. Two-way ANOVA with Sidak’s multiple comparisons post-test. (F) Representative FACS plots and number of *de novo* accumulating CD45.1^+^ WT lymphocytes recovered from WT and *Tlr4*^−/−^ lymph node transplants 6 h after *i.v* cell injection, and LPS *s.c* administration. *n* = 6 transplants/group. Paired *t* test. Numbers at the top of the graphs indicate fold changes in cellularity in *Tlr4*^−/−^ vs. WT transplants. (G and H) Representative division profiles (CTV dilution), number of OT-I (G) and OT-II (H) TCR transgenic cells and their proliferation metrics following recovery from WT and *Tlr4*^−/−^ lymph node transplants 3 days after *i.v* T cell transfer and ovalbumin (OVA) + LPS *s.c* administration. *n* = 5 transplants/group. Paired *t* test. Numbers at the top of the graphs indicate fold changes in OT-I or OT-II cellularity in *Tlr4*^−/−^ vs. WT transplants. Data represent the mean ± SD with superimposed individual data points. n.s, not significant; ∗, *p* < 0.05; ∗∗, *p* < 0.01; ∗∗∗, *p* < 0.001; ∗∗∗∗, *p* < 0.0001.

The partial requirement for TLR4 expression on radio-resistant cells and the preserved localization of CCL21, CXCL13, and VCAM1 expression (Figures 1E and 1F), led us to hypothesize that LN SCs, which in the steady-state are the main producers of these factors,^7^ may be responsible for the enhanced intranodal lymphocyte accumulation observed upon LPS exposure. To evaluate this hypothesis, we analyzed publicly available transcriptomic data from diverse LN populations^43^^,^^44^^,^^45^ for the expression of *Tlr* mRNA transcripts. This analysis demonstrated variegated expression of *Tlr* mRNAs across the populations analyzed (Figure 2A). Notably, *Tlr4* mRNA abundance was higher on blood endothelial cells (BECs), lymphatic endothelial cells (LECs), and fibroblastic reticular cells (FRCs) as compared to immune cells, including B cells and cDCs, which are known to vigorously respond to LPS. In agreement with these data, we could readily detect TLR4 protein expression on CD45^-^gp38^−^CD31^+^ BECs, CD45^-^gp38^+^CD31^+^ LECs, and CD45^-^gp38^+^CD31^−^ FRCs isolated from LNs using flow cytometry (Figure 2B). BECs, LECs, and FRCs also seemed to express all the components required for TLR signaling (Figure S2A). Nonetheless, only FRCs seemed to consistently degrade IkBa and phosphorylate ERK upon LPS exposure *in vitro* (Figure 2C). Whether such cell-specific response relates to differences in the expression of *Lbp* (highest in LECs), *Ly96* (highest in BECs), *Cd14* (highest in FRCs) or other signaling components downstream of TLR4 between the different SC subsets (Figure S2A) remains to be determined. Notwithstanding, it seems that LN SCs may qualitatively and quantitatively translate TLR stimuli distinctly, with FRCs possibly being more prone to produce inflammatory mediators upon LPS stimulation as compared to BECs and LECs.

To determine if the apparent SC-specific behavior to LPS exposure impacts chemokine expression *in vivo*, we again took advantage of publicly available transcriptomic data.^43^ Comparison of SCs sorted from steady-state vs. LPS-treated LNs of WT mice revealed that LPS exposure consistently led to the upregulation of the inflammatory chemokines Ccl5 and Cxcl9 across all SC subsets (Figures S2B–S2D). In contrast, only FRCs upregulated the expression of the homeostatic chemokines Ccl19, Ccl21a, and Cxcl13 (Figure S2D), suggesting these cells to be the cells responsible for lymphocyte accumulation in LPS-exposed LNs. To determine if LPS directly modulates chemokine expression on FRCs, we generated FRC-like SC lines, as reported previously,^46^ from the skin-draining LNs of WT and *Myd88*^−/−^ mice. Stimulation of our cell lines with LPS confirmed that FRCs directly sensed and responded to TLR ligation with increased Ccl19 and Ccl21 transcription in a MyD88-dependent manner (Figures 2D and S2E). Moreover, in transwell assays, LPS exposure of WT, but not *Myd88*^−/−^, cell lines significantly enhanced lymphocyte migration, which could be prevented by inhibition of Gi/o protein-coupled receptors (GCPRs) with pertussis toxin (PTx) (Figures 2E and S2F), thus implying a functional role for LPS-induced, FRC-derived chemokines in lymphocyte migration. To test whether direct TLR ligand sensing by LN SCs could also modulate lymphocyte trafficking *in vivo*, we generated mice in which LN SCs were rendered LPS insensitive through LN transplantation. Briefly, we grafted *Tlr4*^−/−^ LNs in the popliteal fossa of WT recipients, after excision of the host’s endogenous nodes. The resulting transplants were composed of donor-derived LPS-unresponsive *Tlr4*^−/−^ SCs and host-derived LPS-responsive WT hematopoietic cells,^46^^,^^47^^,^^48^^,^^49^ and were virtually identical to the host’s endogenous LNs in terms of cellular composition and spatial organization (Figures S2G and S2H). Despite these features, when injected with LPS, “chimeric” *Tlr4*^−/−^ transplants recruited significantly fewer *i.v* administered CD45.1^+^ lymphocytes within a period of 6 h as compared to WT LN transplants (Figure 2F).

Immune protection against pathogenic insults critically depends on the number of antigen-specific lymphocytes recruited into the immune response.^3^^,^^50^^,^^51^ Hence, given our results, we next asked whether the ability of LN SCs to modulate lymphocyte accumulation into the reactive LN upon LPS sensing would affect the development of adaptive immunity. Analysis of OVA-responsive OT-I and OT-II cells 3 days after OVA + LPS administration revealed a significant reduction in the number of antigen-specific cells participating in the immune response in *Tlr4*^−/−^ *vs.* WT LN transplants (Figures 2G and 2H). This deficit could not be attributed to impaired T cell proliferation as both the percentage of divided precursors and the division and proliferation metrics of the TCR transgenic cells did not differ between the transplants (Figures 2G and 2H). These results, therefore, support the concept that danger/pathogen detection by LN SCs sustains the induction of adaptive immunity, not by interfering with T cell responses/proliferation at the individual T cell level, but rather by acting globally at the level of immediate T cell precursor accumulation into the reactive LN. Increased attraction of CCR7-expressing lymphocytes to the reactive LN would be a temporary mechanism to possibly diversify the naive lymphocyte repertoire locally, given that homeostatic chemokine expression may later in the response be downregulated by lymphocyte-derived factors, such as IFNg.^30^ Taken together, our *in vitro* and *in vivo* data demonstrate the ability of LPS to trigger chemokine expression on LN SCs, in turn stimulating intranodal lymphocyte accumulation and promoting adaptive immunity.

### Fibroblastic reticular cell TLR4 supports T cell responses

The preceding results were obtained in LN transplants. Though OT-I and OT-II T cells proliferated normally within the transplants, suggesting that both antigen drainage to and support for T cell activation/proliferation within the transplants were not compromised, it could be argued that the “healing” inflammatory process and its putative dependence on TLR signaling^52^^,^^53^^,^^54^ may have altered the intra-transplant environment and compromised our observations. To account for these limitations and identify the SC subset(s) responsible for modulating lymphocyte distribution, we took advantage of *Tlr4*^*flox*^ alleles and selected Cre transgenes to achieve SC-specific TLR4 deletion in a non-surgical context. Initially, we used the *Cdh5-Cre.*^*ert2*^ transgene to target BECs (and LECs—Figures S3A–S3C), suspecting that HEVs would be a prime site for modulating trafficking to the reactive LN. Comparison of *Tlr4*^*fl/fl*^ *vs.* *Tlr4*^*fl/fl*^ *x Cdh5-Cre.*^*ert2*^ mice in the steady-state and following LPS administration revealed no differences in LN cellularity and lymphocyte accumulation, however (Figures S3D–S3F). In agreement with these findings, following immunization, intranodal OT-I and OT-II T cell responses in *Tlr4*^*fl/fl*^ *vs.* *Tlr4*^*fl/fl*^ *x Cdh5-Cre.*^*ert2*^ mice were quantitatively identical (Figures S3G and S3H), suggesting that direct LPS sensing by endothelial cells was largely dispensable in our experimental conditions.

**Figure 3:**
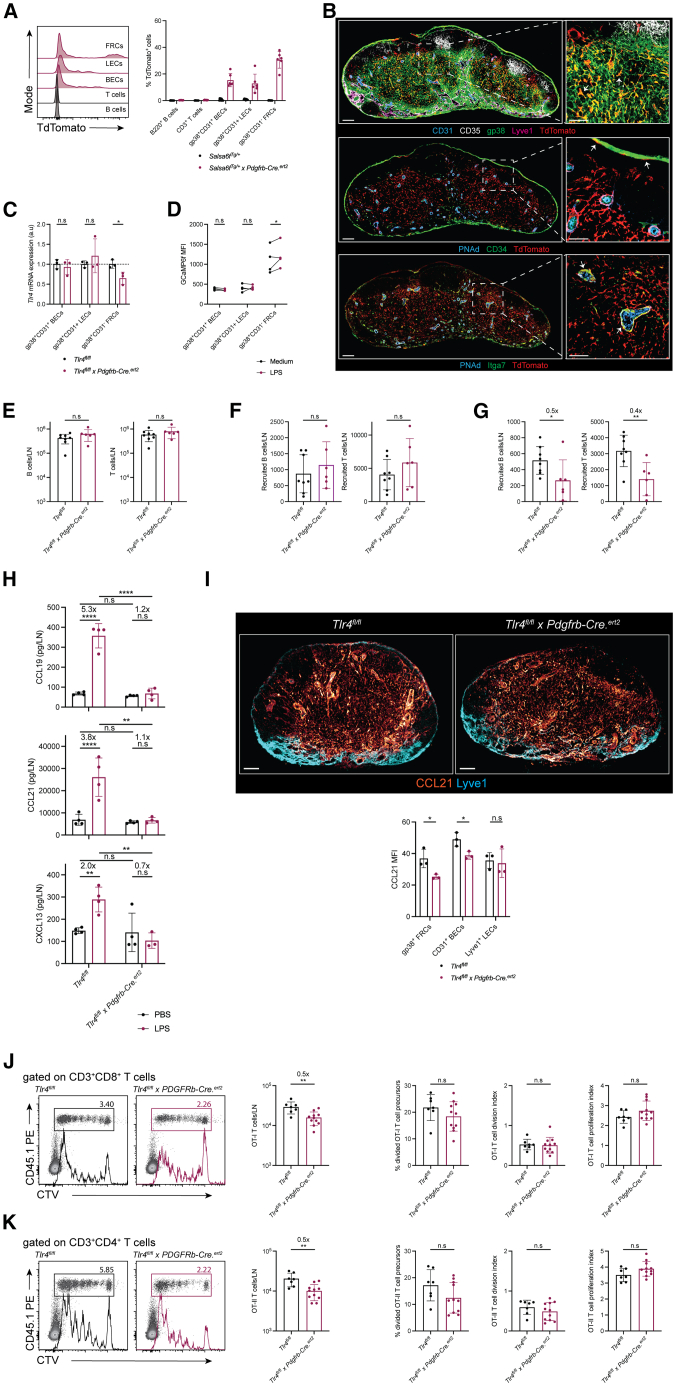
TLR4 expression in FRCs supports T cell responses (A) Representative histograms and frequency of TdTomato^+^ cells in *Salsa6f*^*fl/+*^ and *Salsa6f*^*fl/+*^ x *Pdgfrb-Cre.*^ert2^ mice, 7 days after the last tamoxifen injection. n = 4–6 mice/group. (B) Representative immunofluorescence images of *Salsa6f*^*fl/+*^ x *Pdgfrb-Cre.*^ert2^ lymph nodes, 7 days after the last tamoxifen injection. Arrows in the top inset highlight examples of TdTomato^+^ T cell zone FRCs, arrows in the middle inset highlight TdTomato^+^ CD34^+^ capsular cells and, arrows in the bottom inset depict TdTomato^+^ Itga7^+^ perivascular cells. Scale bars, 200 μm. *n* = 4 mice. (C) *Tlr4* mRNA expression in lymph node stromal cells sorted from the nodes of *Tlr4*^*fl/fl*^ and *Tlr4*^*fl/fl*^*x Pdgfrb-Cre.*^*ert2*^ mice. *n* = 3 mice/group. Two-way ANOVA with Fisher’s multiple comparisons post-test. (D) Quantification of GCaMP median fluorescence intensity prior to and after *ex vivo* LPS stimulation of single cells suspensions of *Salsa6f*^*fl/+*^ x *Pdgfrb-Cre.*^ert2^ lymph nodes. *n* = 4 mice; two-way ANOVA with Fisher’s LSD test. (E) Endogenous lymphocyte numbers in non-LPS exposed auricular LNs of tamoxifen-treated *Tlr4*^*fl/fl*^ and *Tlr4*^*fl/fl*^*x Pdgfrb-Cre.*^*ert2*^ mice. n = 6–8 mice/group. Unpaired *t* test. (F) Number of *de novo* accumulating CD45.1^+^ WT lymphocytes recovered from the non-LPS exposed auricular lymph nodes of tamoxifen-treated *Tlr4*^*fl/fl*^ and *Tlr4*^*fl/fl*^*x Pdgfrb-Cre.*^*ert2*^ recipients 6 h after *i.v* cell injection. n = 6–8 mice/group. Unpaired *t* test. (G) Number of *de novo* accumulating CD45.1^+^ WT lymphocytes recovered from the LPS exposed popliteal lymph nodes of tamoxifen-treated *Tlr4*^*fl/fl*^ and *Tlr4*^*fl/fl*^*x Pdgfrb-Cre.*^*ert2*^ recipients 6 h after *i.v* cell injection, and LPS s.c administration. n = 6–8 mice/group. Unpaired *t* test. Numbers at the top of the graphs indicate fold changes in intranodal cellularity. (H) Intranodal CCL19, CCL21, and CXCL13 expression as quantified by ELISA, 6 h after PBS or LPS administration to tamoxifen-treated *Tlr4*^*fl/fl*^ or *Tlr4*^*fl/fl*^*x Pdgfrb-Cre.*^*ert2*^ mice. *n* = 4 mice/group. Two-way ANOVA with Sidak’s multiple comparisons post-test. (I) Representative immunofluorescence images and quantification of intranodal CCL21 on lymph node stroma cells in the popliteal lymph nodes of tamoxifen-treated *Tlr4*^*fl/fl*^ and *Tlr4*^*fl/fl*^*x Pdgfrb-Cre.*^*ert2*^ mice 6 h after LPS administration. CCL21 signal is depicted in a divergent scale of red to yellow. Scale bars, 100 μm. *n* = 3 mice/group. two-way ANOVA with Sidak’s multiple comparisons post-test. (J and K) Representative division profiles (CTV dilution), number of OT-I (J) and OT-II (K) TCR transgenic cells and their proliferation metrics following recovery from the popliteal lymph nodes of tamoxifen-treated *Tlr4*^*fl/fl*^ and *Tlr4*^*fl/fl*^*x Pdgfrb-Cre.*^*ert2*^ mice 3 days after *i.v* T cell transfer and OVA + LPS *s.c* administration. n = 7–11 mice/group. Unpaired *t* test. Numbers at the top of the graphs indicate fold changes in OT-I or OT-II cellularity. Data represent the mean ± SD with superimposed individual data points. n.s, not significant; ∗, *p* < 0.05; ∗∗, *p* < 0.01.

Hence, we next explored the role of TLR4 expression on FRCs using the *Pdgfrb-Cre.*^*ert2*^ transgene. This transgene caused partial recombination in FRCs — and to a lesser extent also on BECs and LECs, yet without significant loss of *Tlr4* mRNA in the latter cells (Figures 3A–3C). Among gp38^+^ FRCs variable targeting was noted within T cell zone reticular cells (TRCs), with no particular spatial arrangement indicative of selective Cre expression in any of the recently described TRC subsets,^22^^,^^55^^,^^56^^,^^57^ and within CD34^+^ capsular cells and Itga7^+^ perivascular cells (Figure 3B). To test the specificity of the *Pdgfrb-Cre.*^*ert2*^ transgene we used the Salsa6f reporter system, which contains two fluorescent probes—TdTomato that permanently labels Cre expressing cells and GCaMP6f, a Ca2^+^-responsive green fluorescence emitter that allowed us to infer LPS responsiveness. Consistent with our IkBa and pERK measurements (Figure 2C), recording of GCaMP6f activity in TdTomato^+^ cells revealed that only FRCs would flux Ca2^+^ in response to LPS stimulation *ex vivo* (Figure 3D). Notably, whereas TLR4 deletion with the *Pdgfrb-Cre.*^*ert2*^ transgene had no appreciable effects on LN cellularity and LN lymphocyte homing in the steady state (Figures 3E and 3F), it significantly impaired *de novo* lymphocyte accumulation into LPS-draining LNs (Figure 3G); a deficit that correlated with impaired upregulation of CCL19, CCL21, and CXCL13 expression in *Tlr4*^*fl/fl*^ *x Pdgfrb-Cre.*^*ert2*^ lymph nodes (Figures 3H and 3I). Coherently, OT-I and OT-II T cell responses were significantly reduced in *Tlr4*^*fl/fl*^ *x Pdgfrb-Cre.*^*ert2*^ as compared to *Tlr4*^*fl/fl*^ nodes (Figures 3J and 3K). As before, reduced intranodal TCR transgenic T cell numbers were not the result of impaired T cell proliferation. Taken together, our results support a role for direct TLR4 triggering in FRCs as a driver of intranodal lymphocyte accumulation, quantitatively powering developing T cell responses.

### Fibroblastic reticular cell TLR4 supports anti-tumor immunity

**Figure 4:**
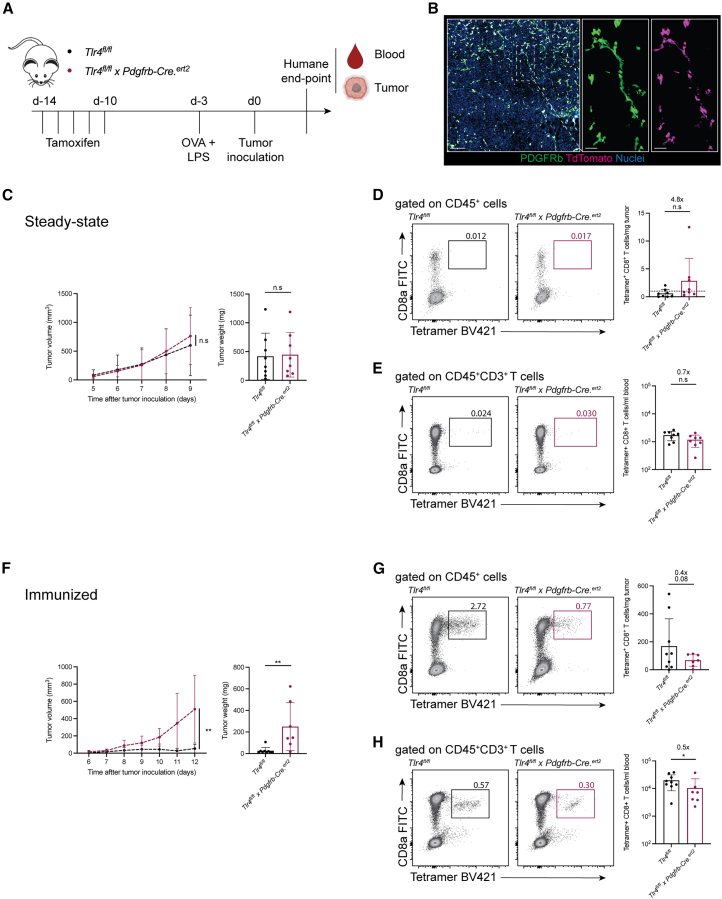
TLR4 expression in FRCs supports anti-tumor immunity (A) Experimental scheme for the induction and assessment of anti-tumor immunity. (B) Representative immunofluorescence images of B16-OVA tumors implanted in tamoxifen-treated *Salsa6f*^*fl/+*^ x *Pdgfrb-Cre.*^*ert2*^ mice. Scale bars, 100 μm; insets = 30 μm. *n* = 3 tumors. (C) B16-OVA tumor volume and weight 9 days post tumor inoculation in non-immunized, tamoxifen-treated *Tlr4*^*fl/fl*^ and *Tlr4*^*fl/fl*^*x Pdgfrb-Cre.*^*ert2*^ mice. *n* = 8 mice/group. two-way ANOVA (left) and unpaired *t* test (right). (D and E) Representative FACS plots and number of tumor-infiltrating (D) or blood-circulating (E) OVA-specific CD8^+^ T cells in non-immunized, tamoxifen-treated *Tlr4*^*fl/fl*^ and *Tlr4*^*fl/fl*^*x Pdgfrb-Cre.*^*ert2*^ mice 9 days post tumor inoculation. *n* = 8 mice/group; Unpaired *t* test. (F) B16-OVA tumor volume and weight 12 days post tumor inoculation in OVA + LPS immunized, tamoxifen-treated *Tlr4*^*fl/fl*^ and *Tlr4*^*fl/fl*^*x Pdgfrb-Cre.*^*ert2*^ mice. n = 7–9 mice/group. two-way ANOVA (left) and unpaired *t* test (right). (G and H) Representative FACS plots and number of tumor-infiltrating (G) or blood-circulating (H) OVA-specific CD8^+^ T cells in OVA + LPS immunized, tamoxifen-treated *Tlr4*^*fl/fl*^ and *Tlr4*^*fl/fl*^*x Pdgfrb-Cre.*^*ert2*^ mice 12 days post tumor inoculation. n = 7–9 mice/group; Unpaired *t* test. Data represent the mean ± SD with superimposed individual data points. n.s, not significant; ∗, *p* < 0.05; ∗∗, *p* < 0.01.

To probe the physiologic relevance of our findings, we proceeded to challenge our mice with B16-OVA melanomas (Figure 4A). In the steady-state, and despite targeting of tumor-associated fibroblast by the *Pdgfrb-Cre.*^*ert2*^ transgene (Figure 4B), both *Tlr4*^*fl/fl*^ and *Tlr4*^*fl/fl*^ *x Pdgfrb-Cre.*^*ert2*^ mice exhibited identical tumor development with limited evidence of ongoing anti-tumor immunity (Figures 4C–4E). In the setting of a prophylactic OVA + LPS “vaccine”, however, *Tlr4*^*fl/fl*^ *x Pdgfrb-Cre.*^*ert2*^ mice exhibited poorer control of tumor growth as compared to *Tlr4*^*fl/fl*^ mice (Figure 4F). This differential behavior correlated with fewer OVA-specific CD8^+^ T cells in tumors (Figure 4G) and circulation (Figure 4H) of *Tlr4*^*fl/fl*^ *x Pdgfrb-Cre.*^*ert2*^ mice, which were induced by vaccination/immunization not only at a distant site from the site of tumor inoculation (hind footpad vs. in-between the scapulae), but also at a separate time point (day −3 vs. day 0). To us, such spatiotemporal separation ruled out possible local effects on tumor-associated fibroblasts, suggesting that an inability to properly recruit T cells into FRC TLR4-deficient LNs at early stages of an immune response limits vaccine efficacy.

## Discussion

Protection against pathogenic insults critically depends on timely, robust, and appropriate effector differentiation of rare antigen-specific lymphocytes. At the single cell level, effector T cell differentiation seems to be a stochastic event, with “hereditary” transmissibility, driven by cell intrinsic properties and environmental constraints collectively dictating TCR signal strength.^16^^,^^17^^,^^18^ Randomness in lymphocytes’ individual behavior is compensated by population averaging of individual cell fates yielding consistent effector programs fit to fight infectious insults.^16^^,^^17^^,^^18^ For the latter to occur, however, sufficiently large numbers of naive T cell precursors must be enlisted into the immune response. Here, pursuing the cellular effects of LPS administration, we showed that optimal T cell recruitment into the immune response requires TLR ligand sensing by LN SCs. Mechanistically, LPS sensing by LN FRCs, not endothelial cells, rapidly increased the expression of the homeostatic chemokines CCL19 and CCL21, which in turn led to immediate intranodal lymphocyte accumulation. Functionally, enhanced lymphocyte accumulation quantitatively powered T cell responses ensuring better outcomes of anti-tumor vaccination efforts.

Notably, our study argues against published evidence suggesting that infection depresses FRC function, leading to reduced homeostatic chemokine and/or cytokine expression.^29^^,^^30^^,^^31^ Instead, our data are consistent with a requirement for MyD88-dependent FRC activation in homeostatic chemokine expression and fat-associated lymphoid cluster formation^36^ and further support an essential role for LN SC activation in intranodal lymphocyte responses.^32^^,^^33^^,^^34^^,^^37^ Bringing all these data into perspective, we suspect that CCL19 and CCL21 expression is acutely upregulated to effectively promote antigen search by exceedingly rare antigen-specific lymphocytes via induction of lymphocyte extravasation, intranodal retention,^58^ and chemokinesis^59^; and that during the proliferative and resolution phases of the immune response, CCL19 and CCL21 expression may need to be ablated to allow optimal T cell proliferation,^60^ prevent secondary T cell priming to unrelated antigens and promote immune resolution.^30^ Therefore, we propose modulation of chemokine expression to be a transient event, similar to inhibition of lymphocyte egress from reactive LNs,^42^ with later aspects of intranodal lymphocyte accumulation being mediated by remodeling of the LN vasculature,^61^^,^^62^^,^^63^ antigen receptor-mediated cellular arrest,^64^^,^^65^^,^^66^ and/or immune cell-derived factors promoting or inhibiting cellular extravasation.^30^^,^^67^ In line with these considerations, we noticed a discrepancy between *Ccl21* mRNA and CCL21 protein levels *in vivo*, suggesting that acute CCL21 upregulation in response to LPS is not driven by transcription and that CCL21 expression may ultimately be decreased, possibly inducing a switch into the overall intranodal migration requirements.^68^ To what extent LPS-induced post-transcriptional/translational modifications^69^^,^^70^ and/or induced secretion^71^ contribute to increased CCL21 expression remains to be determined.

In addition to the upregulation of chemokines we report, it remains possible that FRC TLR4 expression impacts intranodal lymphocyte accumulation by additional mechanisms. One potential mechanism being rapid relaxation of the FRC network to accommodate the incoming lymphocytes.^72^^,^^73^

It is also noteworthy that whereas our data demonstrates that TLR4-expression on endothelial cells does not regulate lymphocyte accumulation into the reactive LN, it does not indicate that endothelial cells are passive spectators in this process. Indeed, the evolutionarily conserved pyrogenic response to infection is known to stimulate the immune response partly by promoting lymphocyte transendothelial migration across HEVs via increased luminal display of CCL21 (and ICAM1) under the influence of IL6 *trans*-signaling.^74^^,^^75^ It is thus likely that endothelial-intrinsic IL6R signaling may act as a gatekeeper to the LN parenchyma by regulating endothelial transcytosis of FRC-produced chemokines.^76^^,^^77^

Finally, co-option of the homeostatic lymphocyte recirculation mechanisms to concentrate immune cells into reactive LNs in response to TLR ligation is arguably a sensible “choice”. On the one hand, it is likely to allow rapid responses without the need to extensively re-wire gene transcription programs on FRCs (transcriptionally and/or epigenetically). On the other hand, by likely promoting preferential recruitment of naive lymphocytes expressing the cognate homeostatic chemokine receptors, it may broaden antigen-specificities at the priming site.

Taken together with data showing that FRCs respond to host-derived factors during viral infections,^37^^,^^52^ human FRCs respond to TLR ligands,^78^ and MRC and FDC function is regulated by TLR signaling,^33^^,^^34^ our current results further bring FRCs to the forefront of immune regulation, highlighting their role as sentinels of infection and their activation status as a critical component in immunity that should be considered when designing novel immune-mediated interventions.

### Limitations of the study

Throughout our study, we used LPS as the TLR ligand under investigation. While we know that the TLR2 agonist zymosan induces a similar response in FRCs, other TLR ligands might induce different responses. Future work is, therefore, needed to elucidate which TLRs are functionally active in FRCs, which responses they induce in isolation and in combination. In our study, we used the *Pdgfrb.Cre.*^*ert2*^ transgene to target FRCs. As highlighted in the manuscript, this transgene targets all fibroblasts, which raises the possibility that some of our findings may be influenced by additional cellular targeting. Finally, it will be important to verify in the future if the FRC response we report on is a general FRC response to infection or the response of a dedicated FRC subset.

## Resource availability

### Lead contact

Requests for information and/or resources should be directed to and will be fulfilled by the lead contact: Reina E. Mebius (r.mebius@amsterdamumc.nl).

### Materials availability

This study did not generate new unique reagents.

### Data and code availability

• Data requests should be directed to and will be fulfilled by the lead contact.
• This paper does not report original code.
• Any additional information required to reanalyze the data reported in this work is available from the lead contact.

## Acknowledgments

We thank Ronald N. Germain for providing feedback on the initial versions of this manuscript. We thank the flow cytometry and microcopy core facilities at the VUmc and the VIB for continuous support. A.P.B. received funding from 10.13039/501100001871Fundação para a Ciência e a Tecnologia (SFRH/BD/33247/2007) and from the Marie-Sklodowska Curie program (grant 898090).

## Author contributions

Conceptualization: A.P.B. and R.E.M.; methodology and investigation: A.P.B. and E.K.; data analysis: A.P.B.; writing: A.P.B. and R.E.M.; funding acquisition: A.P.B. and R.E.M.

## Declaration of interests

The authors declare no competing interests.

## STAR★Methods

### Key resources table

**Table:** 

REAGENT or RESOURCE	SOURCE	IDENTIFIER
**Antibodies**		

Anti-goat IgG (polyclonal; AF555)	ThermoFisher	A21432; RRID: AB_2535853
Anti-rabbit IgG (polyclonal; AF488)	ThermoFisher	A11008; RRID: AB_143165
Anti-rat IgG (polyclonal; AF555)	ThermoFisher	A78945; RRID: AB_2896336
Anti-rat (polyclonal; AF647)	ThermoFisher	A21247; RRID: AB_141778
B220 (6B2; eF615)	ThermoFisher	42-0452-82; RRID: AB_10852702
B220 (clone 6B2; PE.Cy7)	BD Biosciences	552772; AB_394458
B220 (6B2; APC)	ThermoFisher	103212; AB_312997
B220 (6B2; A700)	BD Biosciences	557957; AB_396957
CCL21 (polyclonal; unconjugated)	R&D systems	AF457; 2072083
CXCL13 (polyclonal; unconjugated)	R&D systems	AF470; RRID: AB_355378
CD3 (17A2; BV480)	BD Biosciences	565642; RRID: AB_2739318
CD3e (145-2C11; PE)	ThermoFisher	12-0031-82; AB_465496
CD4 (RM4-5; eF450)	ThermoFisher	17-0031-82; AB_469315
CD8a (53-6.7; eF506)	ThermoFisher	48-0042-82; AB_1272194
CD8a (53-6.7; PE.Cy7)	ThermoFisher	25-0081-82; AB_469584
CD11c (N418; AF488)	Biolegend	117311; RRID: AB_389306
CD31 (390; eF450)	ThermoFisher	48-0311-82; RRID: AB_10598807
CD31 (390; Fitc)	ThermoFisher	11-0311-82; AB_465012
CD34 (RAM34; unconjugated)	BD Biosciences	553731; AB395015
CD35 (8C12; BV480)	BD Biosciences	553775; RRID: AB_10926208
CD45 (30-F11; Fitc)	ThermoFisher	11-0451-82; AB_465050
CD45.1 (A20; PE)	BD Biosciences	553775; AB_10926208
CD45.2 (104; Fitc)	Biolegend	109806; AB_313443
CD45.2 (104; PE)	ThermoFisher	12-0454-82; AB_465678
CD69 (H1.2F3; AF488)	Biolegend	104516; AB_492845
CD169 (Moma-1; AF647)	Bio-rad	MCA947A647; RRID: AB_322322
F4/80 (BM8; eF570)	ThermoFisher	41-4801-82; RRID: AB_2573611
gp38 (8.1.1; eF660)	ThermoFisher	50-5381-82; AB_11151516
H-2kb OVA tetramer BV421	MBL	TB-5001-4
IkBa (MFRDTRK; APC)	ThermoFisher	17-9036-42; AB_2762604
Itga7 (334908; unconjugated)	R&D systems	MAB3518; RRID: AB_2128441
Lyve1 (ALY7; AF488)	ThermoFisher	53-0443-82; RRID: AB_1633415
Lyve1 (ALY7; eF615)	ThermoFisher	42-0433-82; RRID: AB_10804146
PDGFRb (28E1; unconjugated)	CST	3169S; RRID: AB_2162497
phosphoERK (6B8B69; PE)	Biolegend	369506; AB_2629705
PNAd (Meca79; AF488)	ThermoFisher	53-6036-82; RRID: AB_10804391
PNAd (Meca79; SpYG570)	ThermoFisher	120810; RRID: AB_2888808
Ter119 (Ter-119; Fitc)	ThermoFisher	11-5921-82; RRID: AB_465311
TLR4 (MTS510; unconjugated)	AbCAM	ab174606; RRID: AB_307013
VCAM1 (429; unconjugated)	BD Biosciences	553330; RRID: AB_394786

**Critical commercial assays**		

CCL19 ELISA	ThermoFisher	EMCCL19
CCL21 ELISA	Peprotech	900-K132K
CXCL13 ELISA	R&D systems	DY470

**Experimental models: Organisms/strains**		

C57BL/6J (WT CD45.2^*+*^)	Jackson Labs	Jax664
B6.SJL.*Ptprc*^*a*^*Pepc*^*b*^/BoyJ (WT CD45.1^*+*^)	Jackson Labs	Jax2014
B6.B10ScN-*Tlr4*^lps-del^/JthJ (Tlr4^*-/-*^)	Jackson Labs	Jax7227
B6.129P2(SJL)-*Myd88*^tm1.1Defr^/J (Myd88^*-/-*^)	Jackson Labs	Jax9088
B6.129S2-*Ifnar1*^tm1Agt^/Mmjax (Ifnar^*-/-*^)	Jackson Labs	Jax10830
B6.129P2(C)-*Ccr7*^tm1Rfor^/J (*Ccr7*^*-/-*^)	Jackson Labs	Jax6621
B6.129S6-*Rag2*^tm1Fwa^Tg(TcraTcrb)1100Mjb-Ly5a (CD45.1^*+*^ OT-I TCR Tg)	Taconic Farms	Tac2334
B6.129S6-*Rag2*^tm1Fwa^Tg(TcraTcrb)425Cbn-Ly5a (CD45.1^*+*^ OT-II TCR Tg)	Taconic Farms	Tac1896
B6.Cg-Gt(ROSA)26Sor^tm14(CAG-tdTomato)Hze^/J (TdTomato)	Jackson Labs	Jax7914
B6(129S4)-Gt(ROSA)26Sor^tm1.1(CAG-TdTomato/GCaMP6f)Mdcah^/J (Salsa6f Tg)	Jackson Labs	Jax31968
B6.129-*Tlr4*^tm1.1Jke^/J (*Tlr4*^*flox/flox*^)	Jackson Labs	Jax36079
C57BL/6-Tg(*Cdh5*-cre/ERT2)1Rha (*Cdh5-Cre.*^*ert2*^)	Taconic Farms	Tac13073
B6.Cg-Tg(Pdgfrb-cre/ERT2)6096Rha/J (*Pdgfrb-Cre.*^*ert2*^)	Jackson Labs	Jax29684
TdTomato x *Cdh5-Cre.*^*ert2*^	This paper	Jax7914 x; Jax13073
Salsa6f x *Pdgfrb-Cre.*^*ert2*^	This paper	Jax31968 x; Jax29684
*Tlr4*^*flox/flox*^ x *Cdh5-Cre.*^*ert2*^	This paper	Jax36079 x; Jax13073
*Tlr4*^*flox/flox*^ x *Pdgfrb-Cre.*^*ert2*^	This paper	Jax36079 x; Jax29684

### Experimental model and study participant details

#### Mice

The mice used in this study and their origin are listed in the key resources table. All animals were bred under specific-pathogen free conditions and used between 6 and 12 weeks of age. Both genders were used. All experiments were reviewed and approved by the Vrije Universiteit Scientific and Ethics Committees or by the Ethical Commission for Animal Experimentation of Gent University.

### Method details

#### *In vivo* treatments

To evaluate lymphocyte dynamics, mice were treated with ultra-pure lipopolysaccharide (LPS; from *E.coli* 0111:B1; Invivogen) either subcutaneously in the footpad (2.5ug) or intravenously (10ug) with analysis being conducted 6 hours later. In some experiments, lymphocyte migration to and from lymph nodes was manipulated with anti-CD62L antibodies (BioXcell – clone Mel14; 100ug/mouse *i.p*) or FTY720 (Cayman Chemicals; 10ug/mouse *i.p*): in others experiments, 0.25ug of recombinant CCL19 or recombinant CCL21 proteins (R&D Systems) were injected subcutaneously in the footpad. To assess immune responses, mice were challenged with 2.5ug ovalbumin (OVA; Hyglos) and 2.5ug LPS subcutaneously in the footpad with analyses being performed 3 days later. To induce Cre-mediated recombination, mice received 5 consecutive intraperitoneal injections of 2mg tamoxifen (Sigma-Aldrich) diluted in corn oil (Sigma-Aldrich) with all subsequent manipulations being performed 7 days after the last injection.

#### Lymphocyte transfer

Lymphocytes for *in vivo* transfer were isolated from secondary lymphoid organs of donor mice by mechanical disruption through 70 um strainers. Contaminating erythrocytes were lysed with ammonium chloride-potassium. Total lymphocytes were counted and transferred *i.v* without further manipulation. OT-I and OT-II TCR transgenic cells were purified by magnetic isolation (CD8^+^ and CD4^+^ T cell isolation kits from Miltenyi Biotec) and labeled with CTV (Invitrogen) as per the manufacturers’ instructions. Transgenic cells were transferred *i.v*.

#### Bone marrow chimeras

Bone marrow from recipient mice was ablated by exposing animals to two doses of 6 Gy gamma rays. Donor bone marrow was retrieved from the femurs by PBS flushing and injected *i.v* into lightly sedated recipients, which were treated for 4 weeks with neomycin (Merck) to prevent opportunistic infections following irradiation. Experiments were conducted 8 weeks after the reconstitution procedure.

#### Lymph node transplantation

Lymph nodes were transplanted in the popliteal fossa as described.^46^^,^^49^^,^^79^ Briefly, WT recipients were anesthetized and their popliteal lymph nodes removed. Skin-draining lymph nodes from donor mice were then inserted in the vacant space, and the skin tightly stitched. Each recipient mouse simultaneously received one WT and one *Tlr4*^*-/-*^ transplant. In order to minimize possible draining site-specific effects of the transplanted lymph node,^47^^,^^48^^,^^49^^,^^80^ mice received age-, sex- and location-matched transplants. Transplanted lymph nodes were allowed to engraft for 4 weeks before further manipulation.

#### Tumor inoculation

3 days after prophylactic vaccination with 2.5ug OVA + 2.5ug LPS in the footpad, 0.5 x 10^6^ B16.F10-OVA cells were subcutaneously injected between the scapulae. Tumors were measured daily, with volumes calculated according to the formula: (length x width^2^)/2. At sacrifice, tumors were dissected, weighed, and processed for flow cytometry.

#### Stromal cell lines

Lymph node single cell suspensions were obtained by enzymatic digestion with 0.2mg/ml collagenase P (Roche) and 0.1mg/ml DNAse I (Roche) as described by.^81^ To obtain enriched lymph node stromal cell cultures, the resulting single cell suspensions were plated on collagen-coated flasks and cultured in RPMI culture medium (Gibco) supplemented with 10% heat-inactivated fetal calf serum (FCS; Gibco), 2% glutamine (Lonza), 2% penicillin-streptomycin (Lonza) and 50uM 2-mercaptoethanol (Merck). Stromal cells were allowed to adhere to the collagen matrix overnight and subsequently washed to remove non-adherent hematopoietic cells. Subsequent long-term culture and regular fractionation of the cultures resulted in stable, hematopoietic cell-free FRC-like cell lines, as previously described.^46^

#### *In vitro* chemotaxis

The chemotactic potential of our FRC-like stromal cell lines was assessed in 5um-pore transwell systems (Costar). Briefly, stromal cells were cultured in the bottom well of the transwell device and either left unstimulated or stimulated with 100ng/ml LPS for 6 hours. At this time, non-fractionated WT lymphocytes were placed in the upper compartment of the transwell chamber and allowed to migrate toward the bottom compartment for 3 hours. The number and phenotype of the migrated cells was determined by flow cytometry.

#### Flow cytometry and cell sorting

Single cell suspensions for flow cytometry of lymphocyte populations were obtained by mechanical disruption of lymph nodes and tumors. Single cell suspensions for the analysis and sorting of stromal cells were obtained by enzymatic digestion as described above. Cells were surface-stained for 30 minutes in FACS buffer (PBS/2% FCS/2mM EDTA) on ice; intracellular staining was performed for 30 minutes at room temperature following fixation and permeabilization with Invitrogen's Foxp3 fix/perm kit. The antibodies/reagents used are listed in the key resources table. Knock-out cells, fluorescence minus one (FMO) and isotype stains were used to determine detection thresholds. Samples were acquired on either CyanADP (Dako Cytomation), BD LSRII (BD Biosciences) or BD Fortessa (BD Biosciences) flow cytometers and analyzed with FlowJo software (TreeStar). T cell proliferation metrics were determined with FlowJo’s built-in tools. Percentage divided represents the percentage of the initial precursor population that underwent division; the division index represents the average number of divisions that a cell in the overall population, including non-dividing cells, underwent; the proliferation index indicates the average number of divisions that dividing cells experienced. For the analysis of phosphorylated proteins, cultured cells were fixed with 1.6% PFA at the end of the stimulation period before proceeding to staining. For the analysis of LPS-induced Ca2^+^ signaling, stained lymph node stromal cells were maintained in warmed Ca2^+^ containing buffers; samples were acquired for 2 minutes to determine basal GCaMP6f fluorescence, after which 100ng/ml LPS was added and acquisition proceeded for 10 additional minutes. Stromal cell sorting was performed on BD Aria instruments (BD Biosciences).

#### RNA isolation, cDNA synthesis, and real-time PCR

Analysis of gene expression was performed as described previously.^82^ Briefly, for analysis of tissue mRNA transcripts, lymph nodes were harvested and immediately stored in Trizol (Gibco) at -80^o^C. Lymph nodes were homogenized; RNA was precipitated with isopropanol and released from contaminating DNA by DNAse1 (Fermentas Life Sciences) treatment; cDNA was synthesized from 1ug RNA with the RevertAid First Strand cDNA synthesis Kit (Fermentas Life Sciences). To analyze gene expression in *in vitro* cultured cells, cells were lyzed and mRNA trancripts captured by their polyA tail with Roche’s mRNA capture Kit; cDNA was synthesized with the Reverse Transcription system from Promega. To analyze gene expression in sorted cells, samples were processed with Qiagen’s RNeasy Plus Kit as per the manufacturer’s instructions; and cDNA was synthesized with the Reverse Transcription system from Promega. Real-time PCRs were performed on StepOne (Applied Biosystems) or LightCycler 480 II (Roche) real-time PCR systems in the presence of SYBR-green (ThermoFisher). Transcript expression was normalized to the expression of two selected housekeeping genes determined by the geNORM software as the least variable in our experimental conditions.^83^

#### Transcriptomic analyses

Publicly available transcriptomic data obtained by microarray technology originally published by^43^^,^^44^^,^^45^ were downloaded directly from the Immunological Genome Project Consortium website as RMA normalized data. Following log2 transformation of the data, selected gene sets were analyzed with limma using default parameters.^84^ Selected gene sets included TLRs, TLR-related genes as defined by GO terms 35662, 34142 and 34143, and chemokines. Heatmaps were produced with the ComplexHeatmap package.^85^

#### Immunofluorescence and histocytometry

Upon dissection, lymph nodes were fixed in 1% paraformaldehyde overnight and equilibrated in 30% sucrose for 24 hours. Tissues were then frozen in OCT (Sakura Finetek) and cut into sections of 14um. Staining was performed by overnight incubation with the antibodies listed in the key resources table. Images were acquired on a Leica Sp8 microscope equipped with a 20x/0.75NA objective or on a Zeiss 880 microscope equipped with a 25x/0.8NA objective. Images were processed in Imaris software (Bitplane). For the analysis of chemokine and adhesion molecule expression on stromal cells, images were processed for histocytometry, with minor modifications as compared to our previously described protocols.^8^^,^^86^ Briefly, images were segmented using Imaris’ surface object creation module using manually curated thresholds for the detection of the relevant markers for segmentation – CD31 for blood endothelial cells, Lyve1 for lymphatic endothelial cells, gp38 for T cell-zone fibroblastic reticular cells, CD35 for follicular dendritic cells and peripheral CXCL13 for marginal zone reticular cells; as the boundaries of individual stromal cells were not easily discernible, we split the resulting stromal cell surfaces using arbitrary seeding points of 10um radius or in the case of HEVs took each individual HEV as measurement unit. The quantitative data associated with the distinct objects created was then extracted and used to determine average expression values for each object type and sample.

#### Chemokine ELISAs

6 hours after exposure to PBS or 2.5 ug LPS via the s.c route, popliteal lymph nodes were dissected into 0.5mM PMFS (100ul/node) and homogenized by vigorous shaking with metallic beads. Chemokine levels in the lymph node homogenates were assessed by ELISA as per the manufacturers’ instructions – CCL19 (Thermo Fisher), CCL21 (Peprotech), CXCL13 (R&D Systems).

### Quantification and statistical analysis

Statistical analyses were performed with GraphPad Prism (GraphPad Software Inc.). Data are presented as bar graphs indicating the mean ± standard deviation with superimposed individual data points. The number of replicate observations and the specific statistical tests used in each instance are mentioned in the corresponding figure legends. P-values are depicted as n.s, not significant; ∗, p < 0.05; ∗∗, p < 0.01; ∗∗∗, p < 0.001; ∗∗∗∗, p < 0.0001 and were considered significant when < 0.05.
