# Supplementary material for: TLR ligand sensing by lymph node FRCs directs intranodal lymphocyte accumulation to promote immune responses
Source: iScience. 2025 Oct 8;28(11):113734. doi: 10.1016/j.isci.2025.113734 (PMC12597008; doi:10.1016/j.isci.2025.113734)
Supplement: Document S1. Figures S1–S3 [file mmc1.pdf]

iScience, Volume *28*

Supplemental information

TLR ligand sensing by lymph node FRCs directs intranodal lymphocyte accumulation to promote immune responses

Antonio P. Baptista, Eelco Keuning, and Reina E. Mebius

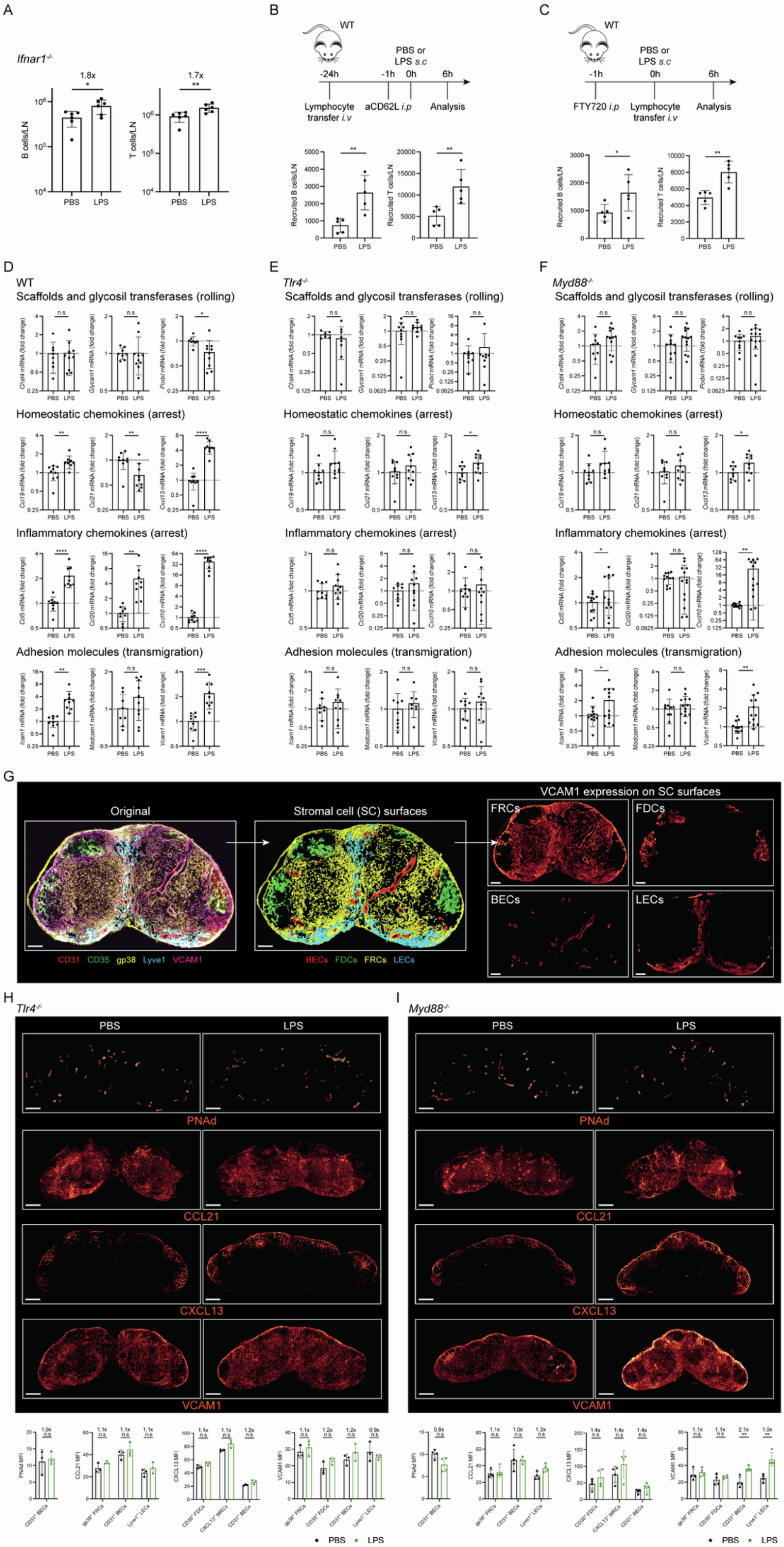
A
Ifnar1-/-
B
WT
PBS or LPS s.c
Lymphocyte transfer i.v
aCD62L i.p
Analysis
C
WT
PBS or LPS s.c
FTY720 i.p
Lymphocyte transfer i.v
Analysis
D
WT
Scaffolds and glycosil transferases (rolling)
Homeostatic chemokines (arrest)
Inflammatory chemokines (arrest)
Adhesion molecules (transmigration)
E
Tlr4-/-
Scaffolds and glycosil transferases (rolling)
Homeostatic chemokines (arrest)
Inflammatory chemokines (arrest)
Adhesion molecules (transmigration)
F
Myd88-/-
Scaffolds and glycosil transferases (rolling)
Homeostatic chemokines (arrest)
Inflammatory chemokines (arrest)
Adhesion molecules (transmigration)
G
Original
Stromal cell (SC) surfaces
VCAM1 expression on SC surfaces
FRCs
FDCs
BECs
LECs
CD31 CD35 gp38 Lyve1 VCAM1
BECs FDCs FRCs LECs
H
Tlr4-/-
I
Myd88-/-
PBS
LPS
PNAd
CCL21
CXCL13
VCAM1

Supplementary Figure 1. **LPS upregulates intranodal chemokine expression.**

A) Lymphocyte numbers in the popliteal LNs of *Ifnar1*$^{-/-}$ mice 6 hours after s.c. injection of PBS or LPS. n = 6 mice/group. Unpaired t-test. Numbers at the top of the graphs indicate fold changes in cellularity as compared to PBS injections.

B) Experimental scheme for the evaluation of the effect of LPS on intranodal lymphocyte accumulation in the absence of lymphocyte entry into lymph nodes. Number of *de novo* accumulating CD45.1^{+} WT lymphocytes recovered from the PBS- or LPS-exposed lymph nodes of mice treated with anti-CD62L antibodies.

C) Experimental scheme for the evaluation of the effect of LPS on intranodal lymphocyte accumulation in the absence of lymphocyte egress from lymph nodes. Number of *de novo* accumulating CD45.1^{+} WT lymphocytes recovered from the PBS- or LPS-exposed lymph nodes of mice treated with FTY720.

D-F) mRNA transcript abundance in the lymph nodes of WT (D), *Tlr4*$^{-/-}$ (E) and *Myd88*$^{-/-}$ (F) mice 6 hours after PBS or LPS administration. Fold changes are calculated over PBS-treated conditions (dotted line). n = 8-10 WT mice/group, 6-10 *Tlr4*$^{-/-}$ mice/group, 11-13 *Myd88*$^{-/-}$ mice/group. Unpaired t-test.

G) Representative histocytometry workflow depicting the original immunofluorescence image, the surface objects created for the different stromal cell populations, and the expression of the signal of interest in the different stromal cell objects. Scale bars = 200um.

H-I) Representative immunofluorescence images and quantification of intranodal PNAd, CCL21, CXCL13 and VCAM1 expression on lymph node stromal cells, 6 hours after PBS or LPS administration to *Tlr4*$^{-/-}$ (H) or *Myd88*$^{-/-}$ (I) mice. Scale bars = 200um. n = 3-4 mice/group. 2-way ANOVA with Sidak's multiple comparisons post-test. Numbers at the top of the graphs indicate fold changes in MFI expression as compared to PBS injections.

Data represent the mean ± SD with superimposed individual data points.

n.s, not significant; *, $p < 0.05$; **, $p < 0.01$; ***, $p < 0.001$; ****, $p < 0.0001$.

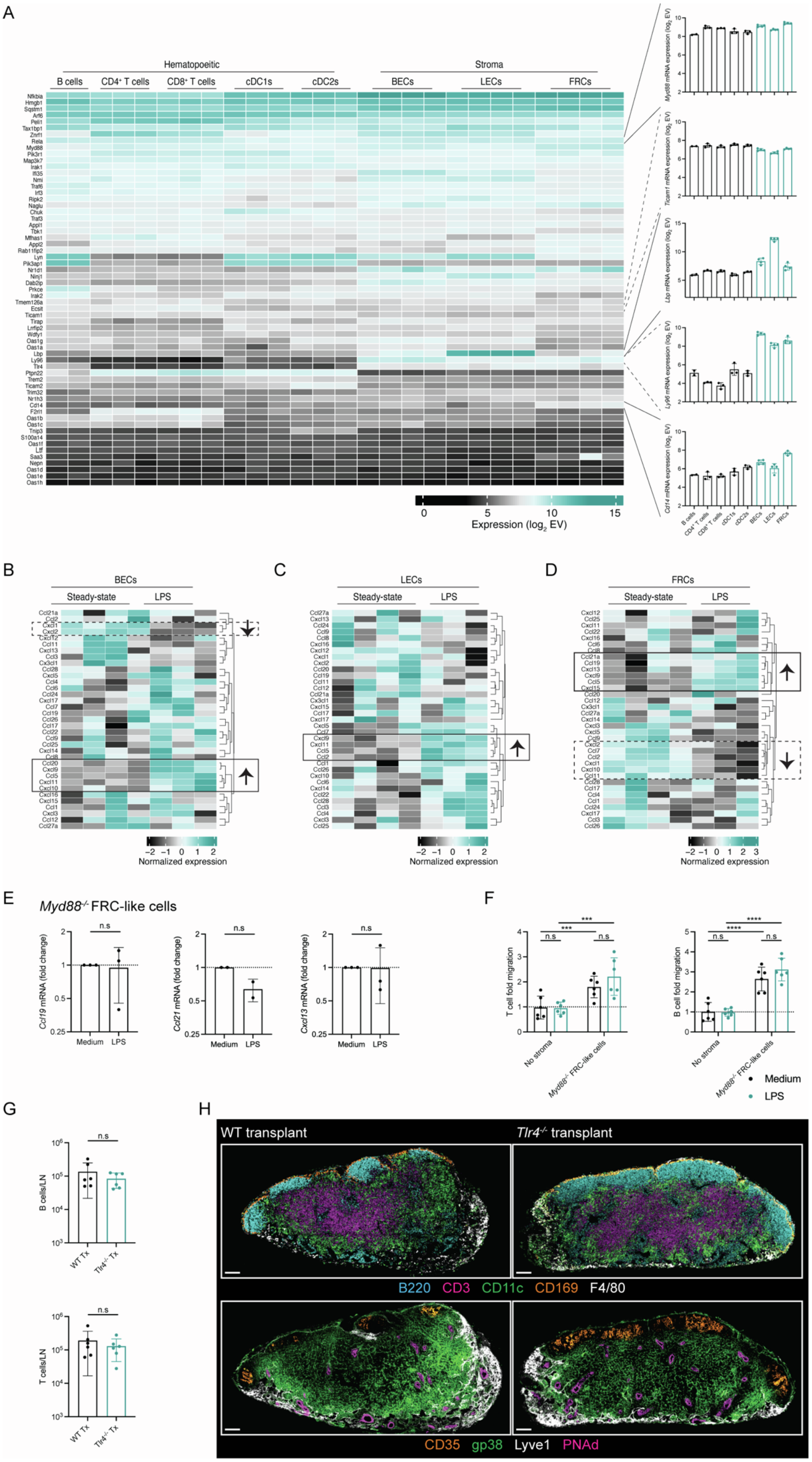
A
Hematopoeitic
Stroma
B cells
CD4+ T cells
CD8+ T cells
cDC1s
cDC2s
BECs
LECs
FRCs
Nfkbia
Hmgb1
Sqstm1
Arf6
Peli1
Tax1bp1
Znrf1
Rela
Myd88
Pik3r1
Map3k7
Irak1
Ifi35
Nmi
Traf6
Irf3
Ripk2
Naglu
Chuk
Traf3
Appl1
Tbk1
Mfhas1
Appl2
Rab11fip2
Lyn
Pik3ap1
Nr1d1
Ninj1
Dab2ip
Prkce
Irak2
Tmem126a
Ecsit
Ticam1
Tirap
Lrrfip2
Wdfy1
Oas1g
Oas1a
Lbp
Ly96
Tlr4
Ptpn22
Trem2
Ticam2
Trim32
Nr1h3
Cd14
F2rl1
Oas1b
Oas1c
Tnip3
S100a14
Oas1f
Ltf
Saa3
Nepn
Oas1d
Oas1e
Oas1h
0
5
10
15
Expression (log2 EV)
Myd88 mRNA expression (log2 EV)
Ticam1 mRNA expression (log2 EV)
Lbp mRNA expression (log2 EV)
Ly96 mRNA expression (log2 EV)
Cd14 mRNA expression (log2 EV)
B cells
CD4 T cells
CD8 T cells
cDC1s
cDC2s
BECs
LECs
FRCs
B
BECs
Steady-state
LPS
C
LECs
Steady-state
LPS
D
FRCs
Steady-state
LPS
−2 −1 0 1 2
Normalized expression
−2 −1 0 1 2
Normalized expression
−2 −1 0 1 2 3
Normalized expression
E
Myd88-/- FRC-like cells
Ccl19 mRNA (fold change)
Ccl21 mRNA (fold change)
Cxcl13 mRNA (fold change)
n.s
Medium
LPS
F
T cell fold migration
B cell fold migration
No stroma
Myd88-/- FRC-like cells
n.s
***
****
Medium
LPS
G
B cells/LN
T cells/LN
n.s
WT Tx
Tlr4-/- Tx
H
WT transplant
Tlr4-/- transplant
B220
CD3
CD11c
CD169
F4/80
CD35
gp38
Lyve1
PNAd

Supplementary Figure 2. **Lymph node transplants are normally organized.**

A) Heat-map analysis of the expression of several mRNA transcripts involved in TLR signaling by cells sorted from skin-draining lymph nodes of WT mice as determined by microarray analysis (log2-transformed data).

B-D) Expression of chemokine mRNA transcripts in lymph node BECs (B), LECs (C) and FRCs (D) from untreated and LPS-treated (12 hours) WT mice. Statistically significant changes are highlighted in boxes.

E) Homeostatic chemokine mRNA transcript levels in FRC-like stromal cell lines of *Myd88*$^{-/-}$ origin cultured in the presence of LPS for 6 hours. Fold changes are calculated over untreated conditions (dotted line). n = 3 independent repeats. Unpaired t-test.

F) Quantification of lymphocyte *in vitro* migration across Transwell membranes toward *Myd88*$^{-/-}$ FRC-like stromal cell lines cultured in the presence of LPS. Fold change is calculated over lymphocyte automigration in conditions without stroma and without LPS. n = 2 independent experiments with triplicate conditions. 2-way ANOVA with Sidak's multiple comparisons post-test.

G) Lymphocyte cellularity in WT and *Tlr4*$^{-/-}$ lymph node transplants. n = 6 transplants/group. Unpaired t-test.

H) Representative immunofluorescence images of WT and *Tlr4*$^{-/-}$ lymph node transplants. Scale bars = 200um. n = 2 WT transplants, 3 *Tlr4*$^{-/-}$ transplants.

Data represent the mean ± SD with superimposed individual data points.

n.s, not significant; ***, $p < 0.001$; ****, $p < 0.0001$.

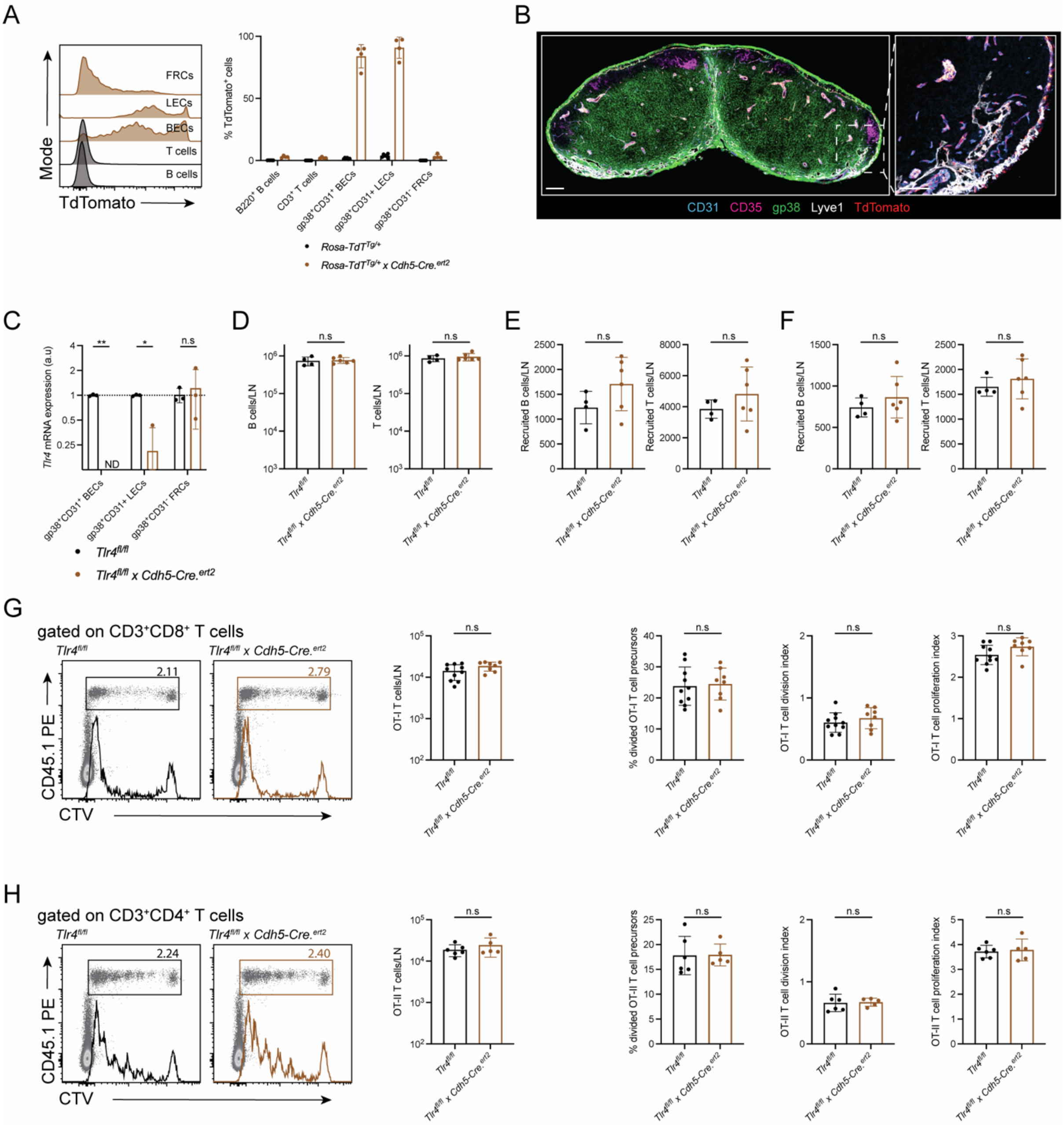

Supplementary Figure 3. **TLR4 expression in endothelial cells does not influence intranodal lymphocyte accumulation.**

A) Representative histograms and frequency of TdTomato+ cells in *Rosa26*$^{TdTomato/+}$ and *Rosa26*$^{TdTomato/+}$ x *Cdh5-Cre.*^{ert2} mice, 7 days after the last tamoxifen injection. n = 4-6 mice/group.

B) Representative immunofluorescence images of *Rosa26*$^{TdTomato/+}$ x *Cdh5-Cre.*^{ert2} lymph nodes, 7 days after the last tamoxifen injection. Scale bar = 200um, inset = 50um. n = 4 mice.

C) *Tlr4* mRNA abundance in lymph node stromal cells sorted from *Tlr4*$^{fl/fl}$ and *Tlr4*$^{fl/fl}$ *x Cdh5-Cre.*^{ert2} mice 7 days after the last tamoxifen injection as determined by RT-PCR. n = 3 samples per cell type and genotype. 2-way ANOVA with Fisher's LSD test.

D) Endogenous lymphocyte numbers in non-LPS exposed auricular LNs of tamoxifen-treated *Tlr4*$^{fl/fl}$ and *Tlr4*$^{fl/fl}$ *x Cdh5-Cre.*^{ert2} mice. n = 4-6 mice/group. Unpaired t-test.

E) Number of *de novo* accumulating CD45.1^{+} WT lymphocytes recovered from the non-LPS exposed auricular lymph nodes of tamoxifen-treated *Tlr4*$^{fl/fl}$ and *Tlr4*$^{fl/fl}$ *x Cdh5-Cre.*^{ert2} recipients 6 hours after *i.v* cell injection. n = 4-6 mice/group. Unpaired t-test.

F) Number of *de novo* accumulating CD45.1^{+} WT lymphocytes recovered from the LPS exposed popliteal lymph nodes of tamoxifen-treated *Tlr4*$^{fl/fl}$ and *Tlr4*$^{fl/fl}$ *x Cdh5-Cre.*^{ert2} recipients 6 hours after *i.v* cell injection, and LPS s.c administration. n = 4-6 mice/group. Unpaired t-test.

G-H) Representative division profiles (CTV dilution), number of OT-I (G) and OT-II (H) TCR transgenic cells and their proliferation metrics following recovery from the popliteal lymph nodes of tamoxifen-treated *Tlr4*$^{fl/fl}$ and *Tlr4*$^{fl/fl}$ *x Cdh5-Cre.*^{ert2} mice 3 days after *i.v* T cell transfer and OVA + LPS *s.c* administration. n = 5-10 mice/group. Unpaired t-test.

Data represent the mean ± SD with superimposed individual data points.

n.s, not significant.
